# A FACS Based Case Study on Two HbE-*β* Thalassaemia Members of a Family, Having Similar Mutational Background

**DOI:** 10.1155/2016/3181937

**Published:** 2016-04-18

**Authors:** Tridip Chatterjee, Suchismita Halder, Amit Chakravarty, Sudipa Chakravarty, Abhijit Chakrabarti

**Affiliations:** ^1^Department of Human Genetics, Institute of Genetic Medicine and Genomic Science, 30A Thakurhat Road, Kolkata 700128, India; ^2^Institute of Genetic Engineering, 30 Thakurhat Road, Kolkata, West Bengal 700128, India; ^3^Crystallography and Molecular Biology Division, Saha Institute of Nuclear Physics, 1/AF Bidhannagar, Kolkata 700064, India

## Abstract

In this report we have tried to explain the reasons behind the difference in the pattern of transfusion requirement between two members of a family with similar *β*-globin mutation. The father and younger son both are HbE-*β*, but the father never had transfusion, whereas the younger son takes transfusion monthly. Mother and the elder son are HbEE without any history of transfusion. *β*-globin mutations of all family members were determined by ARMS-PCR. These were reconfirmed by direct sequencing of *β*-globin gene. Father and younger son were found to be Cod 26 (G-A)/IVS 1-5 (G-C), whereas mother and elder son were found to be Cod 26 (G-A)/Cod 26 (G-A). XmnI sequencing also revealed that all members of the family were CC. Then, flow cytometry study of red blood cells (RBCs) was performed to measure the oxidative stress of the RBCs. This study was also done on the light and dense fractions of the RBC population of the father and younger son. It was seen that the younger son suffers severe oxidative stress, which can be explained by his higher transfusion requirement. From our work, we have established the importance of taking oxidative stress of RBCs into consideration to explain the clinical manifestation and progression of haemoglobin related diseases like thalassaemia.

## 1. Introduction

The HbE-*β* is one of the most common forms of haemoglobinopathies worldwide [1]. The HbE is a splice variant of normal *β* globin protein. This cryptic splice site (related to HbE) is not normally used for mRNA processing. This new splice site competes with the normal splice site and thus produces a protein with a Lys instead of a Glu at position 26 [2]. This variant (HbE) is thus the protein, produced by the said mutation. HbE can be present in both heterozygous and homozygous state, but it can interact with other *β* thalassaemia types to produce HbE-*β* thalassaemia. Several mutants, which interact with the mRNA processing, include abnormalities located in the exon-intron junctions affecting the donor or receptor sites. When one of the nucleotides from the GT/⋯/AG junction is modified the result is a total abolition of the splicing leading to a *β*0 thalassaemic defect. Other mutations have been described within the splicing consensus sequences that seem to play a role in selection of the correct splicing site. HbE is not the mutated protein but the fact is that its *β* subunits are synthesized in too low a quantity to bind all the available alpha chains, resulting in mild *β*+ thalassaemia. The primary clinical importance of HbE trait arises when the *β* ^E^ allele interacts with other *β* thalassaemia mutations leading to moderate-to-severe anaemia known as HbE-*β* thalassaemia [3].

In this paper, we are presenting a typical case which shows that not only can different *β* thalassaemia mutations give rise to a variety of clinical consequences but also the same *β* thalassaemia mutation interacting with *β* ^E^ allele can give a variety of clinical manifestations of the disease condition. Here, two members of the family (the father and the younger son) even after having identical *β* chain mutation show marked difference in terms of clinical severity and transfusion requirement.

## 2. Clinical Representation/Case Report

**Figure 1:**
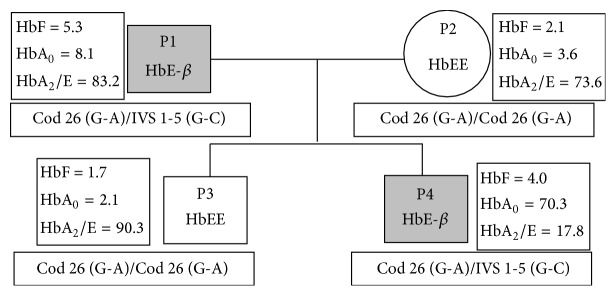
Pedigree of the family along with major clinical and mutational data.

Here, we describe clinical details of a thalassaemic family (Figure 1) showing an unusual and interesting pattern of blood transfusion requirement amongst two members of identical *β* globin mutational background. In the family the father (P1, Ref.  Table 1) is HbE-*β* with no history of blood transfusion. Though his haemoglobin was found to be less than normal (6.8), he never experienced any clinical complications and physiological manifestation of anaemia. The mother (P2, Ref.  Table 1) is HbEE and has no history of blood transfusion. The elder son (P3, Ref.  Table 1) is HbEE, like the mother, and never had blood transfusion requirement. As per classical symptoms of HbEE patients, the mother (P2) and the elder son (P3) both maintain good haemoglobin level and are asymptomatic in terms of expression of anaemic features. The younger son (P4, Ref.  Table 1), who actually came for treatment, is HbE-*β* like his father but is highly anaemic and requires monthly blood transfusion, starting from 8 months of age. We had checked for *α* thalassaemia in all individuals of the family, as it is one of the ameliorating factors, related to clinical manifestation of the disease severity. But no one of the family was *α* thalassaemic, as per our study.

## 3. Materials

Percoll, phosphate buffer saline (PBS) (pH 7.4), and complete protease inhibitor cocktail were purchased from Roche, annexin binding buffer and annexin tagged with FITC were purchased from BD Biosciences, electrophoresis apparatus was purchased from Bio-Rad (Hercules, CA, USA), Diluent and Cymid for Automated Cell Counter (Medonic, EMerck) were purchased from Merck, and *β* thalassaemia short program kit for Variant HPLC was purchased from Bio-Rad (USA). All other reagents, if not mentioned, were purchased locally and were of standard grade.

## 4. Methods

### 4.1. Sample Collection

Peripheral blood samples were collected from every member of the abovementioned family in vials containing 5 mM ethylenediaminetetraacetic acid (EDTA). Written consent was taken from adults and for the children of the family, it was taken from the parents as per the guidelines of the Institutional Ethical Committee. The handling of all human blood samples was carried out in accordance with the guidelines established by the Local Ethical Committee. For the patient (P4), who is on regular blood transfusion, peripheral blood was taken after transfusion gap of 45 days, the maximum gap he can withstand.

Plasma and red blood cells (dense and light fractions) were separated using 75% percoll for the FACS and proteomic studies, as described earlier [4, 5]. Purity of RBCs was checked with antihuman CD235a antibody conjugated with phycoerythrin (CD235a-PE; Serotec, Kidlington, Oxford, UK) and was found to be >99.5%.

### 4.2. Haematological Studies

As per protocol all members of the affected family were evaluated for initial clinical baseline investigations, which are as follows: HbF, HbA_0_, HbA_2_, haemoglobin, Mean Cell Volume (MCV), Mean Cell Haemoglobin (MCH), Mean Cell Haemoglobin Concentration (MCHC), Red Cell Distribution Width (Rdw), and Haematocrit (Hct). The haematologic data (Hb and CBC) were obtained by automatic analysis (Cell Counter: Medonic 530, EMerck) using the manufacturer's protocol. Hb variants (HbA, HbF, and HbA_2_/E) were estimated by HPLC (High Performance Liquid Chromatography) (Bio-Rad, USA) using the manufacturer's protocol. All the samples were also screened for *α* thalassaemia (−*α* ^3.7^ and −*α* ^4.2^ deletion alleles) by GAP-PCR.

### 4.3. *β* Thalassaemia Mutations

DNA was isolated from white blood cells, using a DNA isolation kit from blood (Qiagen). Every member of the family was screened for common *β* thalassaemia mutations of Eastern India [6] like IVS1-1 (G-T), IVS1-5 (G-C), Codon 8/9 (+G), and Fr. 41/42 (−TCTT) along with Codon 26 (G-A) for HbE. The screening was performed by PCR based technique, Amplification Refractory Mutation System (ARMS) as described by Old [7].

### 4.4. XmnI Sequencing

The single-nucleotide polymorphism (SNP) screening of HBG2 gene (1 SNP in NCBI, rs7482144, XmnI site C→T) was done for every individual of this family. To identify the promoter region of ^G^ *γ*-globin, the gene cluster was mapped using Map Draw (sequence analysis software from DNASTAR Inc.). A fragment of the regulatory region of HBG2 gene which contained the* Xmn*I site was identified. The region near the identified promoter sequence was selected to design primers for the* Xmn*I site. The primers were designed using Primer Select (Table 2). PCR amplification of the region of interest spanning the* Xmn*I site was carried out using specific primers (Table 2) in a thermal cycler as per cycling condition, described in Table 4. The PCR components were taken as per Table 3. The amplified products were visualized in 1% agarose gel. The PCR results indicated that the 308 bp fragment was amplified in all samples. For sequencing, the amplicons were usually purified by direct precipitation of PCR products using polyethylene glycol- (PEG-) sodium acetate purification protocol. The finally purified PCR products were sent to commercial sequencing facility.

### 4.5. Flow Cytometry

Blood samples were taken from each member of the family and diluted with PBS (2.7 mM KCl, 1.5 mM KH_2_PO_4_, 137 mM NaCl, and 8.1 mM Na_2_HPO_4_). The diluted sample (~2 mL) was then added in a tube containing 4 mL of 75% percoll without disturbing the layer of percoll. This was then centrifuged at 1200 g for 25 minutes at room temperature. Blood components were separated according to their density. The red blood cell (RBC) dense and light fractions from sample were incubated in presence (test set) and absence (control set) of FITC conjugated annexin V (AV-FITC, BD Biosciences Pharmingen). Flow cytometry of labelled RBCs was performed in FACSCalibur flow cytometer with CellQuest Pro as operating software (Becton, Dickinson & Co., San Jose, CA). The red cell populations were defined by size in forward and side scatter plots. Fluorescence intensities were expressed in logarithmic scale. The control sample incubated without FITC-AV was used to set the region for positive fluorescence such that the fraction of cells with positive (auto)fluorescence was lower than 0.2% of the total. The population of cells labelled with FITC-AV above background was determined from the fraction of cells in this region in excess of that obtained with the (unlabeled) control [8].

## 5. Results

### 5.1. Mutational and XmnI Studies

The HPLC results (Table 1) indicate that the father (P1) and the younger son (P4) are HbE-*β* and the mother (P2) and the elder son (P3) are HbEE. These data were reconfirmed by the results obtained from ARMS-PCR. The genotypes of the four members on the basis of *β* globin mutations are as follows: the father (P1) is Cod 26 (G-A)/IVS 1-5 (G-C), mother (P2) Cod 26 (G-A)/Cod 26 (G-A), elder son (P3) the same genotype as mother Cod 26 (G-A)/Cod 26 (G-A), and the younger son (P4) the same as father, that is, Cod 26 (G-A)/IVS 1-5 (G-C).

The mother (P2) and elder son (P3) are homozygous for HbE; hence no other *β* mutations were found in them, which confirm their homozygous state of HbE. The father (P1) and younger child (P4) have another *β* mutation IVS 1-5 (G-C) along with Cod 26 (G-A), which is responsible for making them compound heterozygous, that is, HbE-*β*.

These mutational studies also confirm that the younger child (P4), who is the main concern of this study, inherits HbE [Cod 26 (G-A)] allele from the mother (P2) and the other mutated *β* [IVS 1-5 (G-C)] allele from the father (P1). Neither –*α* ^3.7^ nor –*α* ^4.2^ deletions were found in any of the individuals of the family by GAP-PCR. Sequencing of the −135 ^G^ *γ*-globin (XmnI region) reveals that all members were CC in genotype. No one was found to be CT or TT.

### 5.2. PS Asymmetry Studies

**Figure 2:**
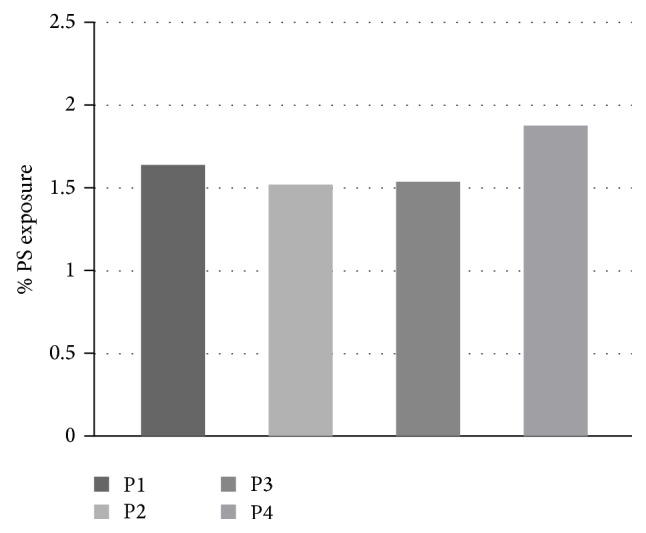
Histogram depicting the % PS asymmetry of the four samples where the *x*-axis denotes the % annexin V-FITC binding or the % PS exposure.

**Figure 3:**
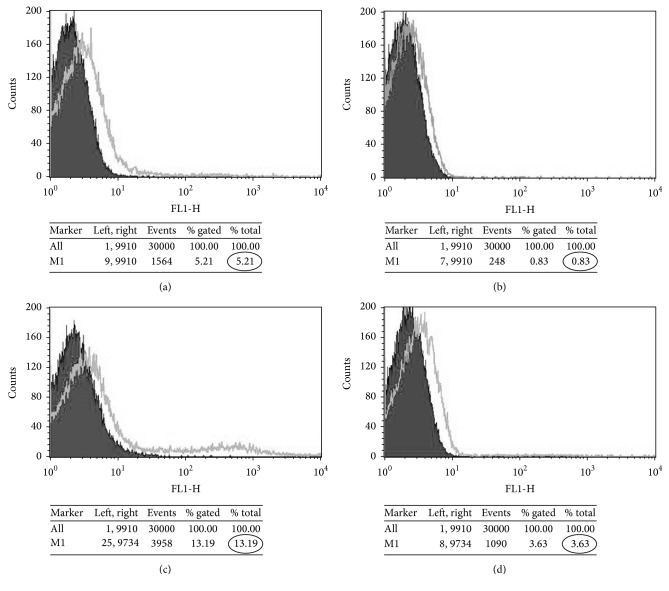
Annexin V-FITC binding of PS in (a) lighter RBC fraction of father, (b) older RBC fraction of father, (c) lighter RBC fraction of son, and (d) older RBC fraction of son.

The percentage of phosphatidylserine (PS) asymmetry of normal RBCs and the patient family was defined by the percentage of annexin V-FITC binding in the young and aged RBC population of peripheral blood. On comparing the percentage PS asymmetries in the total RBC population amongst the four family members, no significant increase could be found (Figure 2), but comparing the young and aged RBCs of the father (P1) and son (P4) showed significant difference (Figure 3) in spite of having the same mutation as stated earlier.

The annexin V-FITC binding data shows clearly that the younger cells show a greater PS asymmetry than the aged RBCs as earlier reported by De Jong et al. [9].  Table 5 shows that the father (P1) having a higher percentage of HbF and HbE shows almost normal PS asymmetry [10]. In case of the son (P4), however, the denser or aged RBC fraction shows a higher percentage of HbF and HbE as compared to the lighter fraction and like the father has lower percentage of PS asymmetry. The lighter RBC fraction of the son (P4) shows a very high percentage of PS exposure and the percentage of HbF and HbE is very low in this fraction.

## 6. Discussion

From genomics studies as reported above, we see that the mother (P2) and elder son (P3) are homozygous E (HbEE) and hence show typical features of this condition. However, the genomics studies did not reveal any significant difference between the father (P1) and younger son (P4) to explain the difference in the pattern of transfusion requirement.

Hence we try to see the condition of their RBCs in the flow cytometry study. When we ran the total RBCs from the four samples, we observed no significant difference in their annexin V-FITC binding, and hence the % PS exposure was not very different. This can be explained by the fact that the mother (P2) and elder son (P3) being homozygous E have RBCs that are almost stable as normal. The father (P1) having no clinical manifestation of the disease also has RBCs showing normal PS asymmetry. However, in case of the younger son (P4), the RBC population observed in our FACS study also showed no significant difference in PS asymmetry.

But interestingly, when the RBCs of the father and the younger son were further separated in light and dense fractions, the HPLC data (Table 5) of these fractions showed that the younger son had a huge fall in the % HbE/A_2_ in the lighter fraction, from which we can assume that the RBC production is somewhat altered. Hence the flow cytometry study of these fractions from the father and the younger son (both carrying the same *β* mutation) shows a marked difference as reported in Table 5. Under normal conditions the PS asymmetry is higher in case of the heavy RBC fractions as compared to the lighter fraction [11]. The opposite trend is usually seen in case of HbE-*β* thalassaemia [10]. Here also we see that the father and son have a higher PS asymmetry in their lighter fraction as compared to the heavier RBC fraction. However, the percentage PS exposure in case of the father is 5.29 (light fraction) and 0.83 (dense fraction) and in case of the son it is 13.34 (light fraction) and 3.76 (dense fraction). The son being highly anaemic shows a huge increase in the PS exposure in both the fractions. This explains the fact that the younger son carries RBCs which are exposed to high oxidative stress and hence undergo severe hemolysis which leads to severe anaemia and transfusion requirement.

## 7. Conclusion

To summarise this case study, we have seen that the genomics study of the family showed that the father (P1) and the younger son (P4) have the exactly identical mutation in their *β* globin gene, but the father has no clinical manifestation, whereas the younger son is severely anaemic and requires blood transfusion almost every month. Unfortunately the genomics study could not shed much light on the reason behind this differential clinical manifestation of disease severity between the father and the son. However, the flow cytometry data and HPLC data after light and dense RBC fractionation clearly showed that the RBCs of the son are undergoing severe oxidative damage and subsequent hemolysis, in spite of carrying the same mutation as the father. From the study, it can also be concluded that the younger son, who requires monthly transfusion, has RBCs which undergo excessive oxidative stress and are very prone to hemolysis. Due to the heavy transfusion taken by the son, a tendency of hypercoagulation is evident in him. It can also be a cause of lowering of HbF percentage, as expected from earlier reports [12].

This study shows the importance of taking oxidative stress of RBC into account and not only the genotype involved in the disease which would provide us with a bit more information on why two people with the same mutation and from the same family will show variation in their clinical manifestation of the disease severity.

## Figures and Tables

**Table 1:** Clinical data of the members of the family in study.

Clinical parameters	P1 (father)	P2 (mother)	P3 (elder brother)	P4 (younger brother)
Haemoglobin (g/dL)	6.8	11.2	11.4	4.1
MCV (fL)	48.5	54.2	50.3	57.0
MCH (pg)	17.2	21.1	21.2	16.6
MCHC (%)	35.4	38.9	42.2	29.2
Rdw (%)	29.0	23.6	22.2	26.4
Hct (%)	19.3	26.8	27.1	14.2
HbA_2_/E (%)	8.1	73.6	90.3	17.8
HbA_0_ (%)	83.2	3.6	2.1	70.3
HbF (%)	5.3	2.1	1.7	4.0
P3 (%)	3.3	5.3	5.1	4.7
History of blood transfusion (BT)	No	No	No	1st BT on 8 months and then on monthly BT

MCV = Mean Cell Volume; MCH = Mean Cell Haemoglobin; MCHC = Mean Cell Haemoglobin Concentration; Rdw = Red Cell Distribution Width; Hct = Haematocrit.

**Table 2:** Primers designed for XmnI sequencing.

Primer	Sequence	Length	Tm	GC%
Forward	AAAATTAAGCAGCAGTATCCTCT	23	48.6	34.8
Reverse	TCCTCCTCTGTGAAATGACC	20	48.6	50.0

**Table 3:** Optimized component concentrations for PCR.

Components	Concentration	Volume/reaction
PCR buffer with MgCl_2_	10x	2.5 *μ*L
dNTPs	2 mM	2.5 *μ*L
Forward primer	10 pm/*μ*L	1 *μ*L
Reverse primer	10 pm/*μ*L	1 *μ*L
Taq polymerase	3 U/*μ*L	0.4 *μ*L
PCR water		15.6 *μ*L
Genomic DNA	50 ng/*μ*L	2 *μ*L
Total		25 *μ*L

**Table 4:** Cycling conditions for PCR.

Condition	Temperature	Time	Number of cycles
Initial denaturation	96°C	5 minutes	—

Denaturation	94°C	30 seconds	30
Annealing	50°C	30 seconds	
Extension	72°C	30 seconds	

Final extension	72°C	10 minutes	—

Final hold	4°C	*∞*	—

**Table 5:** Relative percentage of HbF and HbE in various samples and their relative percentage in dense and light density fractions and their respective PS asymmetries.

Subjects	% HbF	% HbE	RBC fractions	% HbF	% HbE	% PS asymmetry
Father	5.3	8.1	Light	2.6	4.1	5.21
			Dense	8.4	5.2	0.83

Son	4.0	17.8	Light	1.1	3.9	13.19
			Dense	4.5	9.6	3.63
